# Treating a Patient with Your Hands Tied: Acute Chest Syndrome in a Jehovah’s Witness

**DOI:** 10.7759/cureus.7769

**Published:** 2020-04-21

**Authors:** Deepak Vadehra, Tammy Davino, Debapriya Datta

**Affiliations:** 1 Internal Medicine, University of Connecticut Health Center, Farmington, USA; 2 Critical Care, University of Connecticut Health Center, Farmington, USA; 3 Pulmonary Critical Care, University of Connecticut Health Center, Farmington, USA

**Keywords:** acute chest syndrome, sickle cell anemia, jehovah’s witness

## Abstract

Acute chest syndrome (ACS), a vaso-occlusive crisis in patients with sickle cell anemia, is a life-threatening condition and a leading cause of death in these patients. It is treated with analgesics, antibiotics, intravenous fluid, supplemental oxygen (or ventilatory support in severe cases) with simple or exchange transfusion, being the mainstay of therapy. We report a young Jehovah’s Witness (JW) patient with sickle cell anemia who presented with ACS. Her religious beliefs precluded the use of blood products. Despite concomitant hemolytic and aplastic crisis and a hemoglobin nadir of 3.1 g/dL, the patient was treated successfully with supportive care - including mechanical ventilation, sedation, paralysis, and erythropoiesis stimulation - and survived. A maximal supportive strategy consisting of ventilatory support with a high fraction of inspired oxygen, sedation, paralysis, erythropoiesis stimulation, and limitation of blood draws can result in the successful treatment of JW patients who refuse blood products.

## Introduction

Acute chest syndrome (ACS) is a vaso-occlusive crisis of the pulmonary vasculature, occurring in patients with sickle cell anemia. ACS is a life-threatening complication of sickle cell disease (SCD) and is the leading cause of mortality in patients with sickle cell anemia [1]. Early and aggressive interventions are needed to prevent a negative outcome [2]. Standard therapy for ACS includes analgesics, intravenous hydration, and blood transfusions. Simple blood transfusions are typically reserved for ACS cases which are of moderate severity, while more severe cases warrant exchange transfusion [2]. Jehovah's Witness (JW) patients refuse to accept blood transfusions on religious grounds, which makes the treatment of such patients with severe anemia a challenge [3]. Our case highlights the role of the supportive measures in a JW with severe ACS who refuse transfusion of blood products. 

## Case presentation

**Figure 1:**
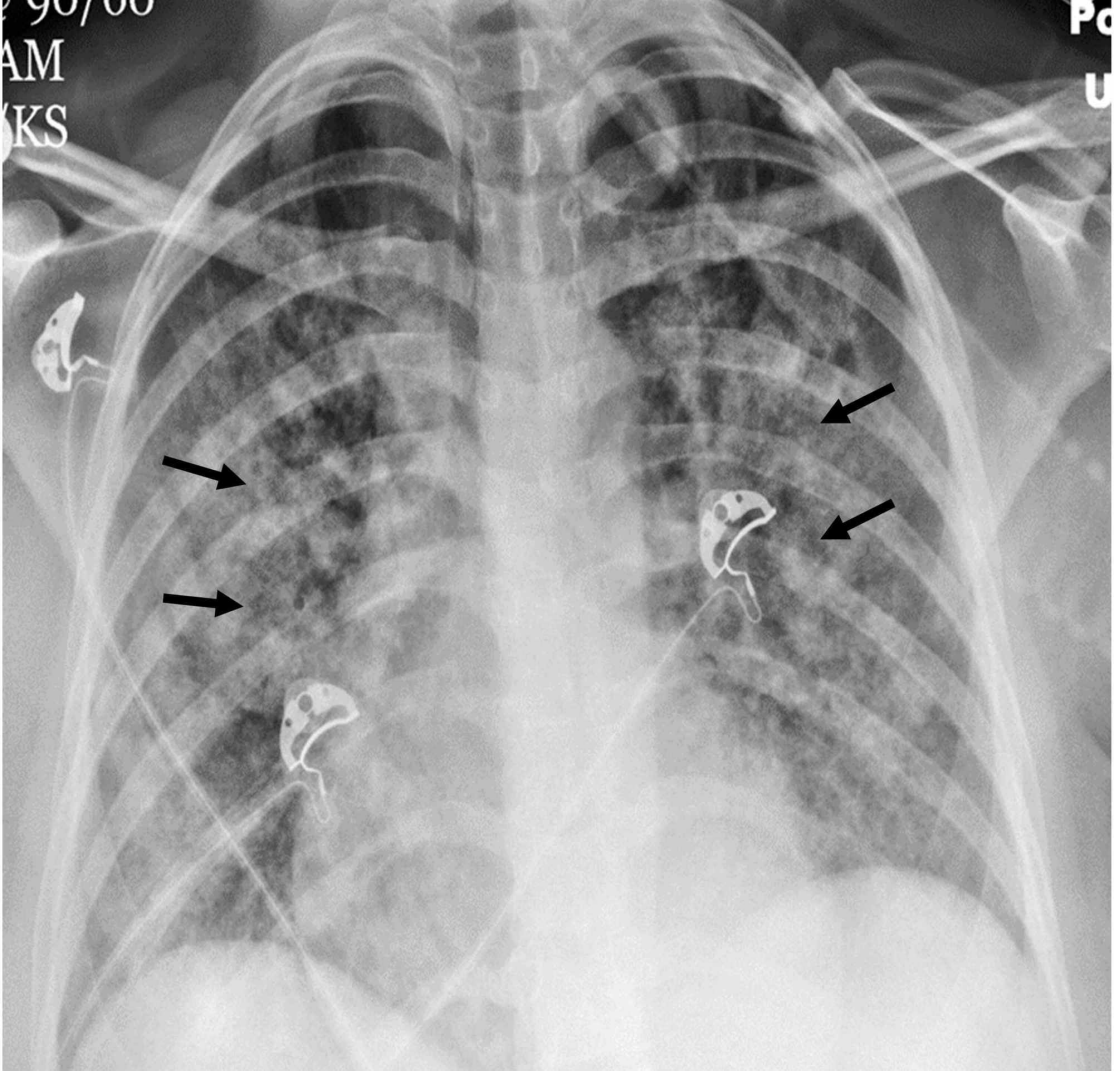
Chest X-ray Chest X-ray (AP view) showing bilateral alveolar opacities suggestive of airspace disease (black arrows)

A 26-year old African-American female, who was a JW with a history of sickle cell disease (SCD; hemoglobin SC), presented to the emergency room with a pain crisis involving the back, arms, legs, and chest. Her exam was normal except for pallor. Her hemoglobin (Hb) at admission was 7.7 g/dL; serum lactate dehydrogenase (LDH) was elevated at 6077 IU, serum haptoglobin was reduced at 24 mg/dl; serum bilirubin and liver enzymes were elevated. She was admitted for an acute pain crisis and treated with intravenous fluids and analgesics. Over the next 24 hours, she developed respiratory distress and was hypoxic. Chest X-ray showed opacities in bilateral lung fields (Figure 1).

**Figure 2:**
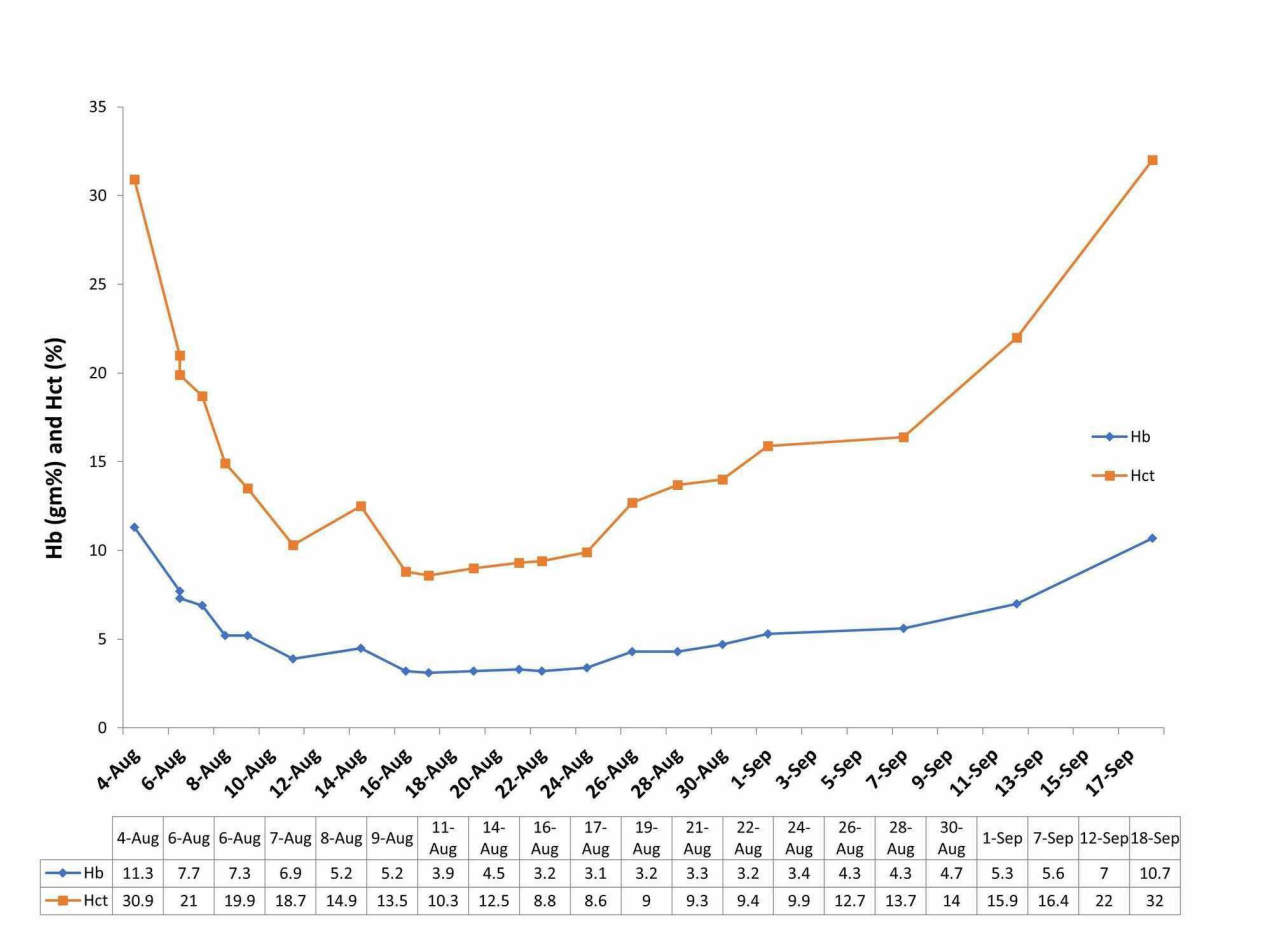
Hemoglobin and hematocrit trend Graphic depiction of the trend of the patient’s hemoglobin (Hb g%) and hematocrit (Hct %) during the hospital stay

The patient was diagnosed as having ACS and was transferred to the intensive care unit (ICU) for further management. The patient’s respiratory status worsened and she was intubated and placed on invasive mechanical ventilation (assist control, with a tidal volume of 6 mL/kg ideal body weight). Her Hb and hematocrit (Hct) continued to decline with a nadir Hb of 3.1 g/dL. To minimize energy expenditure and oxygen consumption (VO_2_), the patient was deeply sedated and paralyzed with intravenous cisatracurium infusion. The patient was placed on 100% fraction of inspired oxygen (FiO_2_), to increase oxygen solubility in the blood and maximize partial pressure of oxygen (pO_2_) in her arterial blood and thereby help oxygen delivery (DO_2_) to the tissues. The FIO_2_ was lowered to 50% after 72 hours to prevent oxygen toxicity. While on 100% FIO_2_, the patient's pO_2_ was 150 mm Hg and O_2_ saturation was 100% on 100% FIO_2_. Though the risks of hyperoxia are well known, in this case, it was outweighed by the risk of cellular anoxia due to impaired oxygen delivery, resulting from a severely reduced Hb, which the hyperoxia aimed to correct. In addition, blood loss was minimized by avoiding daily blood draws and pediatric tubes for samples were used, when needed. The patient was administered erythropoietin to stimulate erythropoiesis in conjunction with vitamin B12, folate, multivitamin, and vitamin C. The patient was also loaded with intravenous (IV) iron and given leuprolide injection to prevent blood loss by suppressing menstrual bleeding. Erythropoietin was stopped after two weeks of daily administration. The paralytic drug was discontinued after 7 days. LDH and haptoglobin levels improved indicating resolution of hemolysis. After two weeks, her Hb and Hct began to show slow but steady improvement (Figure 2), and her chest X-ray demonstrated improvement as well. She was eventually weaned off sedation and successfully extubated. At discharge after a 5-week hospitalization, her Hb and Hct had increased to 7.7 g/dL and 23.6%, respectively, and she was ambulating with normal oxygenation on room air. 

## Discussion

ACS is treated with supportive measures, including intravenous fluids, analgesics, supplemental oxygen, and empiric antibiotics [2]. Transfusion is the mainstay of treatment in moderate to severe cases. Simple blood transfusions are administered in moderate cases to keep Hb ~7g/dL. In severe or moderate cases not responding to conventional supportive care, exchange blood transfusion is required [2].

When blood transfusion is not an option in severe cases of ACS due to religious beliefs, such as in this patient, management can be challenging and the treatment limitation can pose a clinical and ethical challenge to the physician [4]. The physician must face the emotional burden of watching a patient deteriorate and even die, in a preventable setting. JW is a rapidly growing religious group in the western world, with over two million members reported worldwide. About 1000 deaths/year have been reported in JW patients from the refusal of blood transfusion. Their refusal of transfusion of blood products has resulted in the acquisition of knowledge of critical Hb levels in humans and has led to the development of the specialty of “Bloodless Medicine” [3].

For physicians caring for JW patients, understanding and accepting their belief regarding blood transfusions is imperative [4]. They also need to be aware of the medico-legal and ethical aspects of the care of such patients. If a physician is unable to accept not treating such patients based on their belief, it is essential they transfer care to another physician who can do so [4]. To treat JW patients who need blood products, it is essential that treating physicians are aware of therapeutic alternative options that maximize oxygen delivery (DO_2_) to tissues and minimize oxygen uptake (VO_2_) and expenditure in the cells [3-4].

JW patients accept medical and surgical treatment but believe that blood transfusion is forbidden for them based on biblical passages, which they view as ruling out transfusion of whole blood, packed red blood cells (RBCs), and plasma, as well as WBC and platelets. The religion does not absolutely prohibit the use of components such as albumin, immunoglobulins, and hemophiliac preparations; each JW must decide individually if he can accept these. Use of dialysis and cardiopulmonary bypass, as well as intra-operative salvage where the extracorporeal circulation is uninterrupted, is permitted; the physician should consult with the individual patient as to what his conscience dictates [5].

Management of JW patients with severe anemia, be it in the setting of trauma, acute blood loss perioperatively, or ACS, without transfusion of blood products, requires supportive care and the use of alternative therapeutic options that maximize DO2 and minimize VO2 [6-7]. This includes: (i) treatment of hypovolemic shock, if present; (ii) local hemostatic interventions (cauterization, fibrin glue) and use of pro-hemostatic agents such as aminocaproic acid; recombinant FactorVIIa; desmopressin to stop bleeding and high-dose recombinant erythropoietin with iron/B12/folate to stimulate erythropoiesis; (iii) ventilatory support to reduce work of breathing and assist with oxygen delivery. (iv) sedation and neuromuscular blockade to minimize energy expenditure [4,6]. In addition, several experimental therapies have been reported in anecdotal case reports to be successful in the treatment in such patients [6-7].

Experimental therapies that have been used include hyperbaric oxygen (HBO), barbiturate coma, hypothermia and the use of blood substitutes. HBO therapy was performed in a monoplace chamber setting at 2.0 atmospheres absolute for 90 minutes per treatment up to twice daily. The patient received a total of 30 HBO treatments and had sustained improvement in all hemodynamic parameters, RBC volume, and renal and respiratory function [8].

Another case report supports the potential role for barbiturate coma in JW patients who may die of anemia when other modalities of support are not sufficient [9]. A 52-year-old JW with SCD had severe anemia associated with multi-organ failure. Standard therapies to reduce O_2_ demand were unsuccessful. Pentobarbital coma was instituted to reduce cerebral O_2_ consumption on day 3. The patient stabilized and survived to discharge.

Hypothermia has been reported to successfully treat severe blood loss anemia in a JW patient [10]. Following emergent colectomy, the patient had Hb of 4 g/dL with hemodynamic instability. Neuromuscular blockade was instituted with cisatracurium, followed by hypothermia to a target of 32°C. This was continued for 3 days until Hb rose from 4 to 5gm/dL; the patient was then rewarmed. The patient survived to be discharged home one week later [10]. This treatment has not been validated by large scale clinical trials; however, it is a widely available treatment modality, which may be helpful when put into clinical practice in such patients.

Additional experimental therapy with a promise that continues to be developed is the use of blood substitutes, Hb-based O_2_ carriers (HBOC), in the treatment of severe blood loss anemia in JW patients. A JW patient with severe anemia due to GI bleed was treated with 7 units of bovine Hb-based oxygen-carrying compound (HBOC-201). High-dose erythropoietin was also administered daily. Hb levels increased to 7.6 g/dL on day 24 of therapy and the patient survived [11]. Polymerized human Hb has been reported to be successful in treating a JW patient with ACS [12]. In a multicenter, unblinded series, 54 patients with severe anemia (median Hb = 4 g/dL) received standard treatment plus HBOC-201. Twenty-three patients (41.8%) were discharged. No serious adverse event was attributed to HBOC-201 [13].

Blood substitutes being studied include perfluorocarbon (PFC)- based blood substitutes. PFCs are surrounded by a lecithin surfactant in a water-based solution. The first approved O_2_-carrying blood substitute was a perfluorocarbon called Fluosol [14]. It was approved by the FDA in 1989 but withdrawn in 1994 due to adverse effects. Other HbOC currently being studied include HemoPure, which is made of chemically stabilized, cross-linked, purified bovine Hb in a salt solution and dextran-Hemoglobin (Dextran-Hb), a conjugate of the polymer dextran with human Hb [15-16]. A study has reported a large-scale ex vivo production of mature human blood cells using hematopoietic stem cells [17]. Other blood substitutes being developed include hyperbranched polymers (HBP or dendrimer) which are iron-containing porphyrins bonded to a shell and can bind O_2_ reversibly like Hb and biodegradable micelles which are recombinant or polymerized Hb encapsulated within micellar-forming amphiphilic block copolymers and respirocytes which are artificial red blood cells that can emulate the function of its real counterpart with increased efficiency [15-16].

A meta-analysis to assess the safety of Hb-based blood substitutes (HBBSs) in surgical, stroke, and trauma patients, looked at 16 trials involving five different products and 3711 patients in varied patient populations. The meta-analysis found the use of HBBSs to be associated with a significantly increased risk of death and MI, compared to standard blood transfusions. Subgroup analysis of these trials indicated the increased risk was not restricted to a particular HBBS or clinical indication [18]. 

In this case, supportive care was the mainstay of treatment in conjunction with deep sedation and paralysis. The only other case report highlighting a maximal supportive therapy approach similar to ours was employed in a trauma patient in Denmark [19]. This case by no means suggests that minimizing VO_2_ by deep sedation and paralysis and improving DO_2_ by increasing FIO_2_ to achieve O_2_ saturation of 100% should be standard therapy. However, it can serve as a therapeutic option when treatment options are limited in such cases.

## Conclusions

The treatment of JW patients with severe anemia who refuse blood transfusions remains a challenge. With the limited availability of blood substitutes, alternative strategies need to be employed in order to maximize the potential for survival for these patients. Experimental therapies remain anecdotal and have not been validated in clinical trials. While research on blood substitutes continues, current treatment consists of supportive care and experimental therapies such as hypothermia, HBO, deep sedation, pentobarbital coma, and neuromuscular blockade. This case highlights a clinical dilemma where one of the accepted standard therapies of ACS - RBC transfusion - cannot be used based on religious grounds. Our maximal supportive strategy resulted in success for this patient and may help such patients who cannot receive blood products.

## References

[1] Vichinsky EP, Neumayr LD, Earles AN (2000). Causes and outcomes of the acute chest syndrome in sickle cell disease. N Engl J Med.

[2] Melton CW, Johnson H (2006). Sickle acute lung injury: role of prevention and early aggressive intervention strategies on outcome. Clin Chest Med.

[3] Jonsen AR (1986). Blood transfusions and Jehovah's Witnesses. The impact of the patient's unusual beliefs in critical care. Crit Car Clin.

[4] Berend K, Levi M (2009). Management of adult Jehovah's Witness patients with acute bleeding. Am J Med.

[5] (2000). Blood Transfusion Policy for Jehovah’s Witnesses. https://www.watchman.org/articles/jehovahs-witnesses/new-watchtower-blood-transfusion-policy/.

[6] Remmers Remmers, PA PA, Speer AJ (2006). Clinical strategies in the medical care of Jehovah’s Witnesses. Am Journal Med.

[7] Hughes DB., Brant WU, Philip SB (2008). The contemporary approach to the care of Jehovah’s witnesses. J Trauma Acute Care Surg.

[8] Graffeo C, Dishong W (2013). Severe blood loss anemia in a Jehovah’s Witness treated with adjunctive hyperbaric oxygen therapy. Am J Emerg Med.

[9] Wang SW, Badami CD, Deitch EA (2009). The use of barbiturate coma as salvage therapy in a postoperative Jehovah's Witness patient with life-threatening anemia. Am Surg.

[10] Klein MJ, Carter TI, Smith MC (2014). Prophylactic hypothermia and neuromuscular blockade to limit myocardial oxygen demand in a critically anemic Jehovah’s Witness after emergency surgery. J Surg Case.

[11] Gannon CJ., Napolitano LM (2002). Severe anemia after gastrointestinal hemorrhage in a Jehovah's Witness: new treatment strategies. Crit Care Med.

[12] Hogue CW, London MJ, Levy JH (2010). Factors affecting survival after the use of a Hb-based O2 carrier in 54 patients with life-threatening anemia. Anesth Analg.

[13] Lanzkron S, Moliterno AR, Norris E (2002). Hb use in acute chest syndrome: a case report. Transfusion.

[14] Gonzáles ER (1980). The saga of 'artificial blood': Fluosol a special boon to Jehovah's Witnesses. JAMA.

[15] Rousseau GF, Giarratana MC, Douay L (2014). Large-scale production of red blood cells from stem cells: what are the technical challenges ahead?. Biotechnol J.

[16] Giarratana MC, Rouard H, Dumont A (2011). Proof of principle for transfusion of in vitro-generated red blood cells. Blood.

[17] Johnson JL, Dolezal MC, Kerschen A (2009). In vitro comparison of dodecafluoropentane (DDFP), perfluorodecalin (PFD), and perfluoroctylbromide (PFOB) in the facilitation of oxygen exchange. Artif Cells Blood Substit Immobil Biotechnol.

[18] Natanson Natanson, C C, Kern SJ, Lurie P, Banks SM, Wolfe SM (2008). Cell-free hemoglobin-based blood substitutes and risk of myocardial infarction and death: a meta-analysis. JAMA.

[19] Lorentzen Lorentzen, K K, Bjarne K, Jørgen J (2013). Supportive treatment of severe anaemia in a JW with severe trauma. Blood Transfus.

